# Cross-Lagged Analysis of COVID-19-Related Worry and Media Consumption in a Socioeconomically Disadvantaged Sample of Community Adults

**DOI:** 10.3389/fpsyg.2021.728629

**Published:** 2021-12-08

**Authors:** Nadia Bounoua, Shelly Goodling, Naomi Sadeh

**Affiliations:** Department of Psychological and Brain Sciences, University of Delaware, Newark, DE, United States

**Keywords:** stress, anxiety, coping, COVID-19, worry

## Abstract

The COVID-19 pandemic has led to an increase in an array of mental health problems. Previous research has shown that media exposure to stressful situations is often related to anxiety and stress. However, given that most existing work has used cross-sectional designs, less is known about the interplay of media exposure and worry as they unfold during sustained exposure to a collective stressor. The current study examined bidirectional associations between COVID-related worry and media consumption over a three-month period. Participants were 87 community adults, the majority of whom were recruited from communities heavily impacted by the COVID-19 pandemic. For three consecutive months, participants asked to indicate how much time they spent worrying and consuming news about the COVID-19 pandemic on a scale from 1 (“never”) to 5 (“most of the day”). Cross-lagged analyses revealed that Pandemic Worry at Month 1 predicted increases in Pandemic Media Consumption at Month 2, which in turn predicted increases in Pandemic Worry at Month 3. Findings suggest that media consumption may be a maladaptive coping strategy that has the iatrogenic effect of increasing worry. Clarifying the causal associations between anxiety-perpetuating processes and media consumption may have important clinical implications for understanding and treating mental health during health pandemics.

## Introduction

The COVID-19 pandemic has led to an increase in an array of mental health problems (Liu and Liu, 2020). These rates may be even higher within low socioeconomic status populations who are already at an increased risk for mental health problems and less access to resources (Rozenfeld et al., 2020; Rudenstine et al., 2021). Anxiety is a particularly concerning potential outcome during a prolonged health crisis, given that it is associated with sustained, heightened hormonal stress responses that put individuals at risk for poor health outcomes (Nusslock and Miller, 2016). In efforts to better understand behaviors that influence mental health during large scale stressors, previous research has identified that increased media exposure may be a risk factor for developing crisis-related anxiety (McNaughton-Cassill, 2001; Busso et al., 2014; Thompson et al., 2017). This is not surprising, given that media consumption, which broadly refers to the intake of information of news and/or information through various media outlets (e.g., television, cell phones, social media), has been associated with a host of poor outcomes, including persistent negative affect and sleep disruptions (Szabo and Hopkinson, 2007; LeBourgeois et al., 2017). Further, previous research shows that media consumption shapes individuals’ views about current events (Elsass et al., 2014), highlighting the importance of considering media consumption in relation to psychological functioning.

Existing data suggest that media consumption may be a relevant predictor of mental health during the COVID-19 pandemic. Media consumption has increased during the COVID-19 pandemic, with the big four news broadcasters in the United States witnessing a 19% spike in viewing during the week of March 16–22, 2020 (Liu and Liu, 2020). Consistent with past work, research is also showing that COVID-related media exposure is associated with increased mental distress (Riehm et al., 2020; Shabahang et al., 2020). These findings, combined with those from previous public health crises, suggest that exposure to stressor-related media may increase feelings of distress, anxiety, and hopelessness. Unsurprisingly, in one of their earliest reports, the World Health Organization recommended “minimizing watching, reading or listening to news about COVID-19 that causes you to feel anxious…” (Buheji et al., 2020; World Health Organization, 2020).

The tendency to consume media during times of crises may be explained by negative reinforcement models of anxiety. Specifically, an ongoing health crisis, such as the COVID-19 pandemic, introduces a great deal of uncertainty, such as the possibility of illness, loss of employment, and death (Wang et al., 2020). For example, Chen and Hong (2010) found that individuals who experience anxious emotions during a collective crisis might cope by seeking crisis-related information. As they attempt to ease negative emotions that arise in uncertain situations, information-seeking may, in part, provide individuals with a sense of control over the crisis or its impact. Indeed, studies have shown that being informed about the COVID-19 pandemic was associated with virus-related anxiety (Jungmann and Witthöft, 2020).

Staying informed about the COVID-19 pandemic may be one way to cope with pandemic-related worry. Worry, which can be broadly defined as intrusive and excessive concern about future events, is considered a related yet distinct construct from anxiety (e.g., Zebb and Beck, 1998; Segerstrom et al., 2000). Specifically, previous work has distinguished worry, a primarily cognitive manifestation, from anxiety which is characterized by physiological symptoms (Andrews et al., 2010). Thus, although a core feature of anxiety disorders, worry refers to a unique cognitive process in which individual engages in worry as an avoidance-based coping strategy to handle distress (e.g., Borkovec et al., 2004; Andrews et al., 2010; Newman et al., 2013). Persistent engagement in worry-based cognitions has been identified as a predictor of future anxiety disorders across various samples (e.g., Mathews, 1990; Esbjørn et al., 2015; Ryum et al., 2017).

Increased media exposure during the pandemic may represent a potential coping strategy to handle pandemic-related distress. However, this strategy may “back-fire” and actually induce more worries (Witthöft et al., 2018). Indeed, some research has shown that media-related rumination is linked to negative outcomes such as psychological distress and onset of symptoms of trauma disorders (Jenness et al., 2016; Parris et al., 2020). In one three-year longitudinal study, Thompson et al. (2019) found that media exposure to the 2013 Boston marathon bombings positively predicted distress and worry about future negative events. Further, conceptual models of anxiety indicate that although worry may represent a coping strategy in the short-term, worry is associated with increases in negative affect and may sustain distress in the long term (Newman et al., 2013). Taken together, these patterns of findings suggest that while a degree of media consumption during a health pandemic may initially alleviate some pandemic-related distress, an overconsumption of media may inadvertently increase worry through mechanisms of overexposure and rumination.

One limitation of the previous work is the use of cross-sectional designs, which limits the ability to examine these associations over time, rendering impossible the potential to make causal interpretations about the relationship between media exposure and anxiety. One exception is a study by Schmidt et al. (2021) who examined COVID-19 media exposure and COVID-related worry across 21days and found that media exposure predicted next day worry which in turn predicted next day media exposure. These findings provide preliminary evidence that a reciprocal relationship between worry and news consumption exists. However, the extent to which news consumptions and anxiety transact across larger periods of time remains relatively unknown, which is problematic given the sustained nature of the COVID-19 pandemic. To address this gap in the literature, the current study examined bidirectional associations between the degree of COVID-related worry and media consumption over a three-month period in a sample of socio-economically diverse community adults living in the United States. Based on previous work (e.g., Schmidt et al., 2021), we hypothesize that higher levels of COVID-related worry will predict increases in COVID-related media exposure, and that increases in media exposure will be associated with increases in COVID-related worry over time.

## Materials and Methods

### Participants

All participants had previously completed a research study in our laboratory and were invited to complete a virtual follow-up study seeking to examine impacts of the COVID-19 pandemic on mental health. For the initial study, participants were recruited from the community using flyers and online advertisements (e.g., Facebook, Craigslist) and were eligible to participate if they were between the ages of 18–55years old and were able to communicate fluently in English. The first 90 people who responded to this request were enrolled in the study.

Participants for the present study were 87 socioeconomically diverse community adults (M/SD_age_=33.8/9.38years old; 51.2% female), with 55.3% of the sample identifying as Non-Caucasian/White, 23.0% as Black/African-American, 12.6% Asian, and 9.2% reporting some other race. Approximately 17.2% of the sample identified as Hispanic. About half of the participants (53.5%) were working full-time at the time of the study, followed by 16.3% were working part-time, 15.1% were unemployed, 10.5% were students, with the remainder indicating some other employment status (e.g., retired, receiving disability). The median household income fell between the range of $25,000–$49,999. The majority of participants came from communities with COVID-19 positivity and unemployment rates higher than the national average at the time of assessment (Cerron, 2020).

### Measures

All procedures were approved by the University Institutional Review Board (Protocol #‘s: 1073423–17, 1,361,164–1, 1,590,453–6) and followed the ethical standards of the 1964 Declaration of Helsinki. Interested participants provided written consent prior to data collection. For three consecutive months (9/20–11/20), participants completed an online assessment, in which they were asked to indicate: (1) “how much time they spent reading or watching news related to the coronavirus” and (2) “how much time they spent worrying about the COVID-19 pandemic” on a scale from 1 (“never”) to 5 (“most of the day”). The data that support the findings of this study can be made available from the corresponding author upon request.

### Data Analysis

Repeated measures GLM were conducted to examine temporal trends in *Pandemic Worry and News Consumption* across the three time points. These analyses were conducted using SPSS (v.26). To test the central hypothesis, cross-lagged analyses were conducted using Mplus 8.0 (Muthén and Muthén, 2017). Specifically, autoregressive and cross-lagged pathways among constructs over time were specified. Missingness at each timepoint was as follows: Month 1 (.=3; 3.4%), Month 2 (.=7; 8.0%), and Month 3 (.=6; 6.9%). In order to include all participants with at least partial data, missing data were estimated using maximum likelihood estimator with robust standard errors (MLR). Standardized results are reported below.

## Results

### Preliminary Results

**Figure 1:**
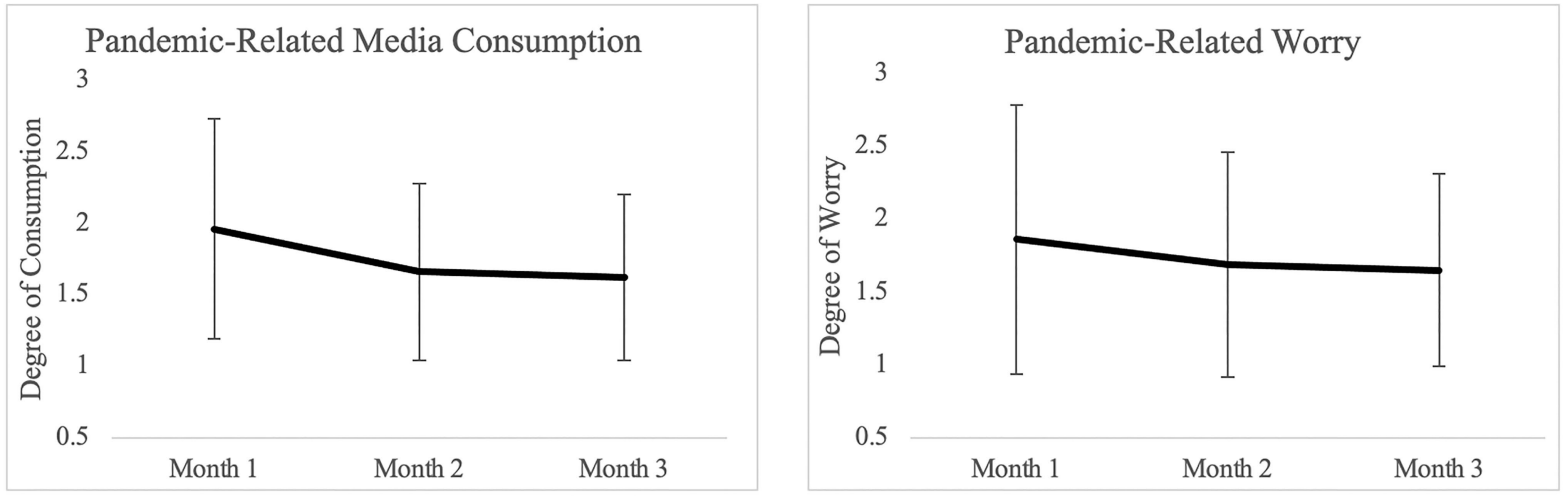
Trends in pandemic-related media consumption and worry across three-month period. Graphs depict means and standard deviations at each time point.

Average trends in these variables over time are depicted in Figure 1. Preliminary analyses indicated that, on average, participants reported less *Pandemic Media Consumption* (*F*=18.89, *p*<0.001) and *Pandemic Worry* (*F*=8.05, *p*=0.006) over the three month period. No significant associations between income or pandemic-related *Media Consumption* and *Worry* were observed at any time point (.’s>0.05). These results indicate that the extent to which respondents worried and consumed news related to the COVID-19 pandemic varied across the three timepoints, allowing for the analysis on potential causal effect of these variables on each other over time.

### Longitudinal Associations

**Figure 2:**
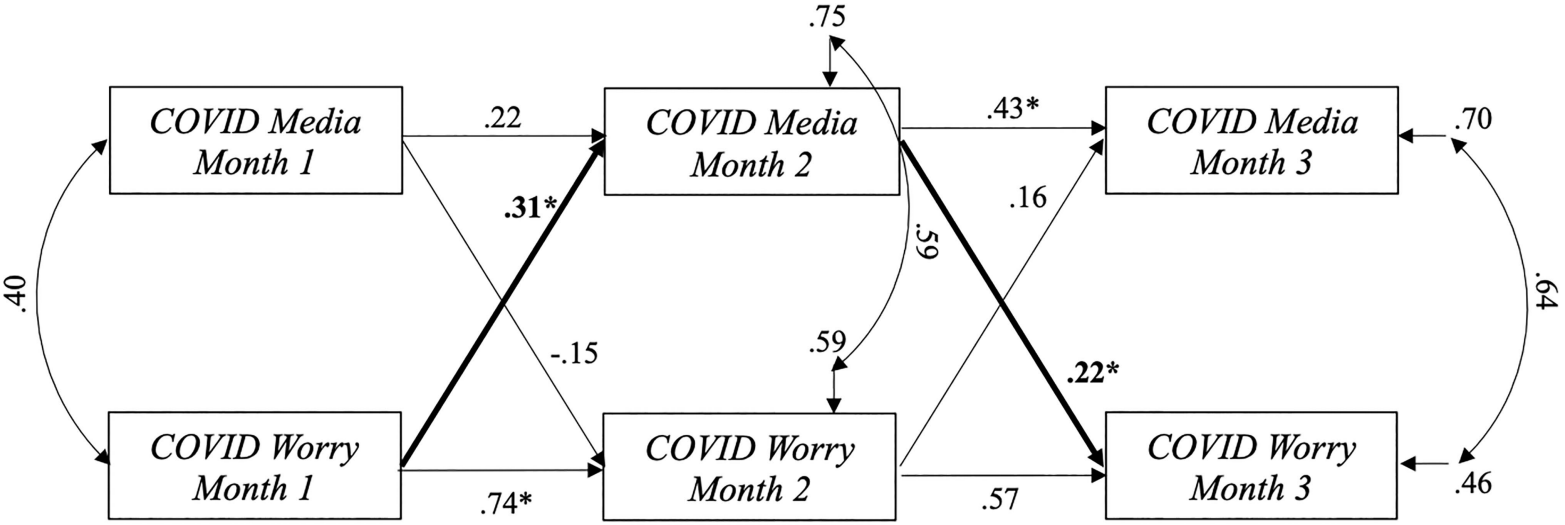
Lagged associations between pandemic-related media consumption and worry over three-month period. *.<0.05. Standardized coefficients are shown. Bolded pathways represent significant cross-lagged associations. $R_{\mathrm{media}}^{2}=0.30$ . $R_{\mathrm{worry}}^{2}=0.54$_._

Results of the cross-lagged analyses are depicted in Figure 2. Analysis of the cross-lagged relationships revealed the data partially supported our hypothesis. Specifically, *Pandemic Worry* at Month 1 positively predicted *Pandemic Media Consumption* at Month 2, indicating higher levels of worry was associated with an increase in the amount of time individuals spent consuming news related to the pandemic over and above the influence of media consumption at Month 1 on Month 2. Further, the cross-lagged path from *Pandemic Media Consumption* at Month 2 to *Pandemic Worry* at Month 3 was significant, indicating greater amount of time spent watching or reading news related to the pandemic was associated with an increase in worry about COVID-19 over and above the influence of worry at Month 2 on Month 3. Unexpectedly, the cross-lagged paths from *Pandemic Media Consumption* at Month 1 to *Pandemic Worry* at Month 2 and from *Pandemic Worry* at Month 2 to *Media Consumption* at Month 3 were not significant. The overall model explained 29.7% variance in *Pandemic Media Consumption* and 54.1% of variance in *Pandemic Worry* at Month 3.

### Parsing Trait and State Pandemic Worry

We conducted secondary analyses to explore whether the findings for Pandemic Worry could be accounted for by individual differences in trait-worry as assessed with the Penn State Worry Questionnaire total score (PSWQ; Meyer et al., 1990). First, bivariate correlations showed that trait worry was not associated with Pandemic Worry at any timepoint (.’s=−0.14-0.12, *p*’s>0.05), suggesting that these variables are assessing two distinct constructs. Next, to test whether the cross-lagged effects of Pandemic Worry were driven by trait-levels of anxiety, we reconducted the cross-lagged analysis including trait worry as a covariate at each time point. Results revealed that all significant pathways remained significant, and no new findings emerged. Taken together, these results suggest that the observed findings cannot solely be accounted for by trait-level factors and instead reflect fluctuations in state COVID-related worry over time.

## Discussion

The current study sought to examine how pandemic-related media exposure and worry mutually influence each other over a three-month period. Results revealed a bidirectional association between worry and media consumption related to COVID-19, such that the degree of pandemic-related worry at baseline was associated with increases in consumption of pandemic-related media a month later. Further, media consumption at month 2 was associated with more pandemic-related worry at month 3, over and above pandemic-related worry at the month 2 assessment.

Past research during other public health crises has shown that consuming stressor-related media leads to heightened stress response and increases in anxiety and other mental health symptoms (Busso et al., 2014; Thompson et al., 2017). Studies during the COVID-19 pandemic thus far have reported similar findings across various samples (e.g., Garfin et al., 2020; Liu and Liu, 2020). While these findings are informative, the temporal associations between these processes as they unfold over time are still unknown. To our knowledge, only one other study has examined these links over time and found support of cyclical daily associations between pandemic-related news exposure and worry (Schmidt et al., 2021). Given the longevity and sustained nature of the current pandemic, the current study extends this research by showing that the bidirectional relationships between media consumption and worry persist across larger intervals of time.

Findings suggest that consuming pandemic-related news may represent a maladaptive strategy to cope with the ongoing pandemic stress, which has the iatrogenic effect of increasing worry. Previous research suggests that individuals are more likely to watch stressor-related media to cope with ongoing stress during times of crises (Liu and Liu, 2020). For example, some research has found that anxious individuals may be more likely to cope with widely broadcasted terrorist attacks by seeking out information on social media (Jin et al., 2016). The current findings are in line with these previous studies and suggest that worry may induce greater news consumption during the COVID-19 pandemic. Given that worry has been identified as a risk factor for the development of anxiety disorders (e.g., Mathews, 1990; Esbjørn et al., 2015; Ryum et al., 2017), these findings suggest that elevated engagement in worry during the COVID-19 pandemic may lead to increases in anxiety-related psychopathology.

One important consideration is that some degree of worry and media consumption may be adaptive in the context of global crises, like the COVID-19 pandemic. Indeed, one study found that flu-related fear mediated the relationship between media exposure and engagement in preventative measures during the 2009 H1N1 outbreak (Zhang et al., 2015). In the context of the COVID-19 pandemic, some degree of exposure to pandemic-related media may be necessary to remain up to date on changes in health protocols and policies, which are critical to “stop the spread” of the virus. However, perseveration on the topic through media consumption is problematic for emotional well-being. This would be consistent with research characterizing persistent worry as a maladaptive, avoidance-based coping strategy to handle distress (e.g., Borkovec et al., 2004; Andrews et al., 2010; Newman et al., 2013). Thus, the public should be cautioned against over-exposure to media related to the pandemic, because it can actually lead to poorer emotional health, which may compromise immune system function (Nusslock and Miller, 2016), which is particularly detrimental during a pandemic.

The current study has several strengths, including a three-time point longitudinal design which allowed for temporal causality to be inferred, the inclusion of a socioeconomically disadvantaged sample of community adults, and a timely question that furthers understanding of mental health during the ongoing COVID-19 pandemic. However, the findings should be interpreted in light of the limitations of the study design. First, like other studies on the impact of the pandemic on mental health (e.g., Schmidt et al., 2021), the measures used to assess worry and media consumption during the COVID-19 pandemic in the current study consisted of single items, which limited our ability to evaluate the psychometric properties of these indices. The rationale for using single items was to reduce burden on participants. Given the limitations inherent in using single-item assessments, we purposefully repeated the measurement over multiple timepoints to provide some index of the reliability of the relationships under investigation. Still, additional research is required to ascertain the construct validity and reliability of the measures used. Second, data collection occurred between October and December 2020, which precludes our understanding of how these processes may have manifested earlier in the pandemic. Third, although previous studies with similar sample sizes have conducted CLPMs (e.g., Juengst et al., 2017), our sample size may have limited our power to detect important effects. Future research should aim to examine these associations in larger samples, particularly among individuals drawn from highly impacted communities, to replicate the findings. Findings could be bolstered by continued investigations into the interplay between media consumption and anxiety-based processes that may continue during, and perhaps, after the COVID-19 pandemic. Similarly, implications of the current are limited to individuals with similar characteristics to the sample used in this study. Generalizability of the findings would be bolstered if future studies examined the associations between media exposure and psychological distress using nationally representative samples, as recently done by other studies examining mental health trajectories in large, national samples (Li and Wang, 2020; Gong et al., 2021). Future research should also aim to examine trends in pandemic-related worry and media consumption to further understand the dynamic nature of these constructs over time. Such analyses are necessary to further elucidate the complex associations between media consumption and anxiety processes as they unfold during collective stressors.

Given the sustained nature of the COVID-19 pandemic, research is needed to identify proximal processes that are contributing to the spike in mental health problems. The goal of the current study was to examine the longitudinal associations between COVID-related news consumption and worry during a three-month period of the COVID-19 pandemic. Despite potential large-scale, public health benefits of media coverage during a pandemic, results revealed that individuals with COVID-related worry engaged in more future news coverage, which then increased their worry. Findings suggest that news consumption, which has increased during the COVID-19 pandemic, may be a useful intervention target during mental health treatment.

## Data Availability Statement

The raw data supporting the conclusions of this article will be made available by the authors, without undue reservation.

## Ethics Statement

The studies involving human participants were reviewed and approved by Institutional Review Board, University of Delaware. The patients/participants provided their written informed consent to participate in this study.

## Author Contributions

NB contributed to conceptualization, data curation, formal analysis, project administration, roles/writing – original draft, and writing – review and editing. SG contributed to roles/writing – original draft, and writing – review and editing. NS contributed to data curation, formal analysis, funding acquisition, investigation, methodology, and writing – review and editing. All authors contributed to the article and approved the submitted version.

## Funding

This research was supported in part by the National Institute of Mental Health awarded to NB [1F31MH120936-01A1]. The Institute had no role in the study design, collection, analysis, or interpretation of the data, writing the manuscript, or the decision to submit the paper for publication.

## Conflict of Interest

The authors declare that the research was conducted in the absence of any commercial or financial relationships that could be construed as a potential conflict of interest.

## Publisher’s Note

All claims expressed in this article are solely those of the authors and do not necessarily represent those of their affiliated organizations, or those of the publisher, the editors and the reviewers. Any product that may be evaluated in this article, or claim that may be made by its manufacturer, is not guaranteed or endorsed by the publisher.
